# Decoding perceived risks in online healthcare services: a safety–trust model based on grounded theory

**DOI:** 10.3389/fdgth.2026.1762306

**Published:** 2026-03-19

**Authors:** Chenlei Lin, Shijie Xiong

**Affiliations:** School of Public Administration and Law, Fujian Agriculture and Forestry University, Fuzhou, China

**Keywords:** grounded theory, online forums, online healthcare platforms, online healthcare services, perceived risk, user comments, web-crawling

## Abstract

**Introduction:**

The rapid rise of online healthcare services (OHSs) in China has improved access to medical information and services while creating new uncertainties related to quality, security, and trust. This study aims to deepen the understanding of perceived risk in OHSs and provide empirical guidance for digital health governance, patient safety strategies, and the development of trustworthy online medical platforms.

**Methods:**

Using a grounded theory approach, we analyzed 106,162 user-generated comments collected from four major online health forums to identify the multidimensional structure and temporal evolution of perceived risk in OHSs.

**Results:**

The analysis produced 22 categories consolidated into four domains: professional-medical, institutional–transactional, technical–data, and relational-emotional , which together constitute a perceived safety–trust model. The model explains how professional uncertainty triggers institutional anxiety, technological fragility heightens perceived vulnerability, and emotional detachment amplifies distrust. The salience of perceived risks shifted over time from professional concerns (2015–2018) to institutional and technical issues (2019–2022), and later to relational-emotional concerns (2023–2024).

**Discussion:**

These findings refine the mechanisms underlying perceived risk in OHSs and show that user trust depends on professional competence, technological reliability, institutional transparency, and empathetic communication.

## 1 Introduction

### 1.1 Background

The rapid advancement of Internet technologies has fundamentally reshaped healthcare delivery worldwide (1). Online healthcare services (OHSs) encompass various Internet-enabled health activities, including real-time consultations, diagnostic support, prescription management, and chronic disease monitoring (2, 3). The coronavirus disease (COVID-19) pandemic accelerated this transformation, leading to the widespread adoption of online healthcare platforms (OHPs) (4). The expansion of OHSs in China has been particularly remarkable. By December 2024, the number of online healthcare users in China reached 418 million, accounting for 37.7% of all netizens (5), and 78.3% of these users accessed OHSs more than three times annually (6). Its unprecedented scale and diversity of users make China a critical context for exploring the opportunities and risks of digital healthcare development.

OHSs offer several advantages. By leveraging digital technologies and rapid data processing, these services reduce the dependence on traditional infrastructure and improve the efficiency of healthcare resource allocation (7, 8). They help relieve hospital overcrowding, shorten waiting times, and enable convenient remote management of chronic conditions (9–11). In addition, OHSs enhance accessibility and continuity of care (12), facilitate patient-doctor communication (13), and contribute to the overall public well-being (14). However, the rapid expansion of OHSs has generated multiple sources of uncertainty that may diminish user trust and sustained engagement (15). These risks primarily manifest in issues such as privacy and service quality fluctuations (16, 17), data security vulnerabilities, and declining professional trust (18–20), reflecting patients’ growing concerns about the reliability and integrity of online healthcare systems.

### 1.2 Prior work

The concept of perceived risk provides a valuable analytical framework to systematically interpret these uncertainties. Originally developed in consumer psychology, perceived risk describes the uncertainty and potential for negative outcomes faced by individuals during decision making (21). Bauer introduced the concept of capturing perceived uncertainty (22). Dowling et al. defined this as the likelihood and severity of possible adverse consequences when purchasing a product or service (23). Jacoby and Kaplan classified perceived risk into five categories: financial, performance, physical, psychological, and social (24). Peter and Tarpey later added time risk, forming the classical six-type framework (25). In the context of online service, perceived risk refers to potential losses associated with achieving desired outcomes when using digital services (26).

Recent studies have extended this framework to include digital health. Deng, et al. (27) defined perceived risk in mobile health (mHealth) as “one's perception of uncertainty in the use of mHealth services and the severity of its consequences.” Although originally proposed for mHealth, this conceptualization provides a valuable reference for understanding perceived risk in broader digital healthcare environments, including OHSs, where interactions depend on digital interfaces, data exchange, and remote medical judgment.

Deng, et al. (27) identified four primary dimensions of perceived risk: privacy risk, performance risk, legal concerns, and trust. Privacy risk reflects users’ concerns about unauthorized access to or misuse of personal medical information (28–30). Performance risk is the extent to which individuals doubt the ability of online medical technologies and services to deliver accurate and reliable healthcare outcomes (27). It captures user uncertainty regarding the diagnostic accuracy and service effectiveness, particularly when physical examinations and face-to-face interactions are limited (31, 32). Legal concern refers to individuals’ concerns about inadequate legal protection and unclear regulatory enforcement in online healthcare contexts (33). Trust can be understood as the perceived credibility and integrity of the digital platform and medical professionals behind it (34–36).

Beyond these dimensions, the commercial nature of OHSs introduces additional sources of perceived risks. Given that many OHSs, such as Good Doctor Online and JingDong Health (37), are operated by profit-oriented enterprises, commercial incentives may intensify perceived risks through excessive marketing, opaque pricing, and diminished professional credibility (38). Collectively, these factors constitute a complex risk landscape that shapes patient trust in and willingness to engage with OHSs.

Despite the growing interest in OHSs, most existing studies have examined individual aspects such as service quality, privacy protection, or patient trust; few have systematically applied the perceived risk framework to this domain. Furthermore, prior research relies predominantly on structured surveys or predesigned scales (39–41), which may overlook emergent, context-specific risks shaped by evolving technologies and user experiences. This gap highlights the need for data-driven and inductive approaches that can capture patients’ perceived risks as they naturally emerge from real-world interactions with OHPs.

### 1.3 Aim

Based on the above analysis, this study adopted an inductive data-driven approach that integrates web crawling with grounded theory. Specifically, we analyzed large-scale user-generated comments from four major online healthcare forums on Baidu Tieba: Good Doctor Online, Good Doctor, JD Health, and Ping An Good Doctor. These forums host active discussions in which patients share personal experiences, evaluate online consultations, and address concerns about service quality and safety. Such patient-generated content offers authentic and diverse insights into user perceptions and behaviors, free from the constraints of structured questionnaires and researcher interventions.

Through grounded theory analysis, this study systematically identified and conceptualized the dimensions of perceived risk emerging from real-world patient discussions, thereby revealing how these risks are formed, structured, and expressed in online healthcare contexts. The findings may advance the theoretical understanding of perceived risk in OHSs and provide empirical guidance for digital health governance, patient safety strategies, and the development of trustworthy online medical platforms.

The objectives of this study were as follows:

(1) To identify and conceptualize the dimensions of patient-perceived risk in OHSs using large-scale user-generated data collected via web crawling.
(2) To examine whether and how these perceived risk dimensions evolved over time, reflecting the dynamic nature of patient risk perception during the development of OHSs.
(3) To construct an empirically grounded conceptual framework explaining how the different dimensions of the perceived risk function interact within an OHS environment.

## 2 Methods

### 2.1 Data source and collection

In this study, we used data collected from four major online health communities on Baidu Tieba, one of the most active discussion platforms in China. These communities are directly affiliated with three of the largest OHPs in the country (3, 42). Specifically, Good Doctor Online operates two official forums—Good Doctor Online Bar and Good Doctor Bar— that serve as patient communities for sharing medical experiences and evaluating doctors. JD Health has an active forum named the JD Health Bar, and Ping An Good Doctor operates the Ping An Good Doctor Bar. Together, these four forums represent diverse online health communities where patients exchange experiences, discuss medical services, and express concerns regarding the quality, safety, and trustworthiness of OHSs.

A Python-based web-crawling program was developed to collect all publicly available posts and comments from the four selected forums. The primary dataset covered 10 years from January 2015 to December 2024, encompassing the rapid expansion and institutionalization of OHSs in China. Additional data from 2014 and January–August 2025 were included for supplementary validation to enhance the comprehensiveness of the analysis. It is important to note that the primary dataset is complete and representative of the study's core period (2015–2024). The inclusion of additional data from 2014 and January–August 2025 was not due to any incompleteness of the primary dataset, but rather to ensure broader temporal coverage, capture both early-stage trends, and include the most recent developments. This supplementary data provides a more comprehensive view of the evolution of online health community discussions over time. It was incorporated to validate the trends observed in the primary dataset and to confirm that the identified categories and concepts were consistent across different time periods. By doing so, the additional data further strengthens the reliability and consistency of the risk-identification framework.

To ensure data relevance, only posts explicitly describing users’ online healthcare experiences, perceptions, or risk evaluations were retained. Irrelevant, duplicate, or purely commercial content was removed by using a combination of automated filtering and manual reviews. After data cleaning, the final corpus contained 106,162 valid user comments, including 103,781 comments from January 2015 to December 2024 used for the main analysis and 2,381 comments from 2014 and January–August 2025 used for supplementary validation.

### 2.2 Analytical approach

#### 2.2.1 Research method

We employed Strauss and Corbin's grounded theory, a qualitative methodology widely used to generate theories about phenomena through the systematic and inductive analysis of empirical data (43). This grounded theory seeks to uncover and explain the underlying mechanisms of social phenomena using rigorous analytical techniques such as coding (44).

This approach is particularly effective in examining complex, culturally embedded, and context-dependent processes, as it emphasizes theory generation that emerges directly from raw data rather than relying on preexisting frameworks (45). In accordance with established grounded theory procedures, the analytical process in this study comprised three iterative stages (46). During open coding, user comments were carefully examined to identify the key concepts and initial categories that emerged from the data. Axial coding involved exploring the relationships among these categories to cluster them into broader, higher-level constructs. Finally, selective coding focused on integrating the main categories into core theoretical constructs that revealed the primary dimensions of patient-perceived risks and their interconnections. These three stages of coding were conducted using Nvivo11 software, which significantly enhanced the efficiency and accuracy of the analysis, allowing for systematic categorization and identification of relationships within the large dataset.

Consistent with the principles of grounded theory, data collection and analysis were conducted iteratively. Supplementary data were incorporated to further examine the stability and temporal applicability of the emerging categories. Insights generated during initial coding informed subsequent sampling, categorization, and analysis through a constant comparative process, ensuring that all categories remained grounded in the data and guiding the gradual refinement of theoretical propositions (43).

#### 2.2.2 Saturation testing

To verify the robustness and completeness of the developed risk-identification framework, multiple procedures were implemented at different stages to ensure analytical consistency and methodological reliability. During the data analysis phase, initial coding was conducted by one researcher and independently reviewed by another. Any discrepancies were discussed through several iterative meetings until consensus was reached; when disagreement persisted, a senior researcher adjudicated the final decision.

A total of 2,381 newly collected patient comments from 2014 and January–August 2025 were incorporated into a validation corpus and systematically cross-checked against the previously established analytical framework. Validation analysis was conducted through a combination of Nvivo11-assisted coding and iterative researcher review. The software facilitated data organization, code retrieval, and comparison across datasets, while substantive judgments regarding category relevance and conceptual novelty relied on researcher interpretation.

Importantly, the validation process remained open to the emergence of new concepts or categories, in accordance with grounded theory principles. Each comment in the validation corpus was carefully examined to determine whether it could be meaningfully assigned to existing categories or whether it suggested the need for new conceptual labels. Although some comments introduced new expressions or contextual nuances, these were analytically interpreted as empirical elaborations or contextual extensions of existing categories, rather than analytically distinct new concepts. No new categories or inter-category relationships emerged that required modification of the established framework.

Code saturation was reached when no new categories appeared in the validation corpus, and meaning saturation was confirmed when additional data no longer elaborated category properties or altered relationships among categories (47). The derived categories were further cross-validated against results from word frequency analysis and relevant literature to ensure internal conceptual coherence.

In addition, three domain experts—senior scholars with established research experience in digital health, health services, and qualitative research—reviewed the coding memos, category structure, and analytical framework. Their independent assessments confirmed that no new codes or categories emerged from the validation corpus and that the relationships among existing categories remained stable. The supplementary data were fully accommodated within the established theoretical framework, indicating that the proposed risk-identification model had reached theoretical saturation.

**Figure 1:**
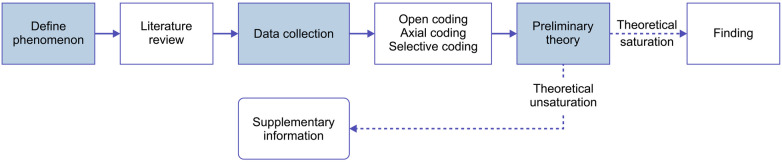
Research procedure based on the grounded theory.

The analytical process, including the coding stages, category refinement, and assessment of theoretical saturation, is presented in Figure 1.

### 2.3 Ethics considerations

The authors did not seek institutional review board approval as this study did not involve human participants or interventions. All analyzed data were publicly available and contained no identifiable personal information.

## 3 Results

### 3.1 Overview of data distribution

**Figure 2:**
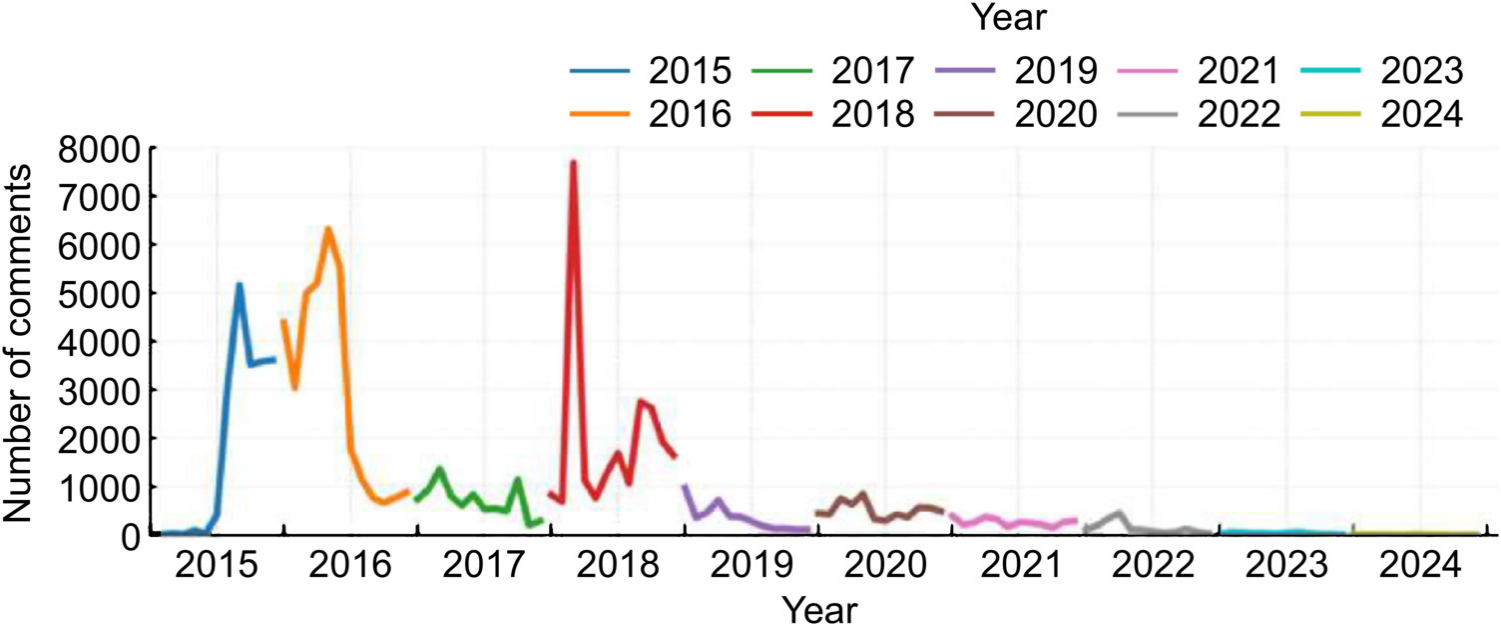
Number of posts related to OHSs from 2015 to 2024.

A total of 103,781 posts related to online healthcare discussions were collected from January 2015 to December 2024 (Table 1). The annual and monthly distributions of posts revealed three distinct temporal phases (Figure 2).

**Table 1:** Number and proportion of posts related to OHSs from 2015 to 2024.

Month	2015	2016	2017	2018	2019	2020	2021	2022	2023	2024
	*n* (%)	*n* (%)	*n* (%)	*n* (%)	*n* (%)	*n* (%)	*n* (%)	*n* (%)	*n* (%)	*n* (%)
January	14 (0.1)	4,385 (12.4)	743 (8.7)	829 (3.4)	974 (22.9)	448 (7.3)	397 (12.4)	106 (6.1)	13 (2.8)	8 (7.1)
February	11 (0.1)	3,041 (8.6)	946 (11.1)	688 (2.9)	359 (8.4)	430 (7.0)	216 (6.8)	195 (11.2)	65 (14.0)	17 (15.0)
March	37 (0.2)	4,995 (14.1)	1,368 (16.0)	7,685 (31.9)	470 (11.0)	758 (12.3)	263 (8.2)	340 (19.5)	58 (12.5)	12 (10.6)
April	20 (0.1)	5,202 (14.7)	809 (9.5)	1,137 (4.7)	722 (17.0)	627 (10.2)	377 (11.8)	457 (26.2)	44 (9.5)	11 (9.7)
May	104 (0.5)	6,318 (17.8)	608 (7.1)	759 (3.2)	392 (9.2)	850 (13.8)	326 (10.2)	119 (6.8)	46 (9.9)	3 (2.7)
June	30 (0.2)	5,547 (15.6)	843 (9.9)	1,282 (5.3)	379 (8.9)	331 (5.4)	168 (5.2)	108 (6.2)	31 (6.7)	16 (14.2)
July	424 (2.2)	1,783 (5.0)	534 (6.3)	1,694 (7.0)	281 (6.6)	290 (4.7)	268 (8.4)	79 (4.5)	47 (10.1)	22 (19.5)
August	3,190 (16.2)	1,151 (3.2)	547 (6.4)	1,066 (4.4)	187 (4.4)	432 (7.0)	256 (8.0)	47 (2.7)	69 (14.9)	8 (7.1)
September	5,168 (26.2)	769 (2.2)	493 (5.8)	2,755 (11.4)	134 (3.1)	370 (6.0)	223 (7.0)	66 (3.8)	44 (9.5)	7 (6.2)
October	3,515 (17.8)	666 (1.9)	1,150 (13.5)	2,623 (10.9)	132 (3.1)	571 (9.3)	148 (4.6)	127 (7.3)	20 (4.3)	2 (1.8)
November	3,584 (18.2)	770 (2.2)	205 (2.4)	1,926 (8.0)	115 (2.7)	565 (9.2)	269 (8.4)	67 (3.8)	16 (3.4)	4 (3.5)
December	3,613 (18.3)	879 (2.5)	290 (3.4)	1,644 (6.8)	113 (2.7)	488 (7.9)	289 (9.0)	35 (2.0)	11 (2.4)	3 (2.7)
Total	19,710 (100.0)	35,506 (100.0)	8,536 (100.0)	24,088 (100.0)	4,258 (100.0)	6,160 (100.0)	3,200 (100.0)	1,746 (100.0)	464 (100.0)	113 (100.0)

Each cell reports the monthly number of posts (n) and their proportion (%), which represents the share of posts in that month relative to the total number of posts in the corresponding year. The last row shows the total annual values.

During the early stage (2015–2018), posting activity increased rapidly, with noticeable peaks in 2016 and 2018. This trend corresponds to the rapid expansion of OHPs in China, where users actively engaged in discussions about their online healthcare experiences on open forums, such as Baidu Tieba, reflecting exploratory attitudes toward emerging digital health services.

The middle stage (2019–2022) coincided with the COVID-19 pandemic, during which OHSs became vital substitutes for in-person healthcare services. Although the absolute number of posts decreased compared to pre-pandemic peaks, online discussions remained vibrant, reflecting the normalization of OHSs and growing user familiarity with OHPs. Thus, the moderate posting volume during this period indicates a behavioral shift from exploratory to routine utilization rather than a decline in public interest.

In the late stage (2023–2024), the number of posts dropped substantially. This decline does not imply diminished public concern but rather reflects broader platforms and behavioral transformations. As user activity gradually migrated from open forums, such as Baidu Tieba, to app-integrated environments (e.g., WeChat mini-programs and official telemedicine apps), fewer posts were generated in public spaces. Moreover, with the maturation of digital health ecosystems, users have increasingly turned to in-app evaluation systems and platform-specific feedback channels for communication, leading to fewer discussions in public forums.

Overall, the dataset spans a full decade from 2015 to 2024 and captures the long-term evolution of public engagement with OHSs in China. Although the posting volume declined after 2022, this trend reflects the natural transformation of online communication patterns rather than a sampling bias. The 10-year time span ensured sufficient temporal coverage and data representativeness, offering empirical support for analyzing the temporal dynamics of patient-perceived risks in subsequent analyses.

### 3.2 Identification of perceived risk

Before formal coding, a frequency-based word cloud analysis was conducted to visualize the most frequently occurring lexical items and condensed terms in user comments. First, the comments were preprocessed through standard text-cleaning procedures, including the removal of stop words, punctuation, and non-informative tokens, as well as the normalization of semantically equivalent terms. Word frequencies were then calculated across the entire dataset, and the resulting frequency distribution was visualized in the form of a word cloud, providing a coarse-grained, intuitive overview of discussion patterns across the dataset.

**Figure 3:**
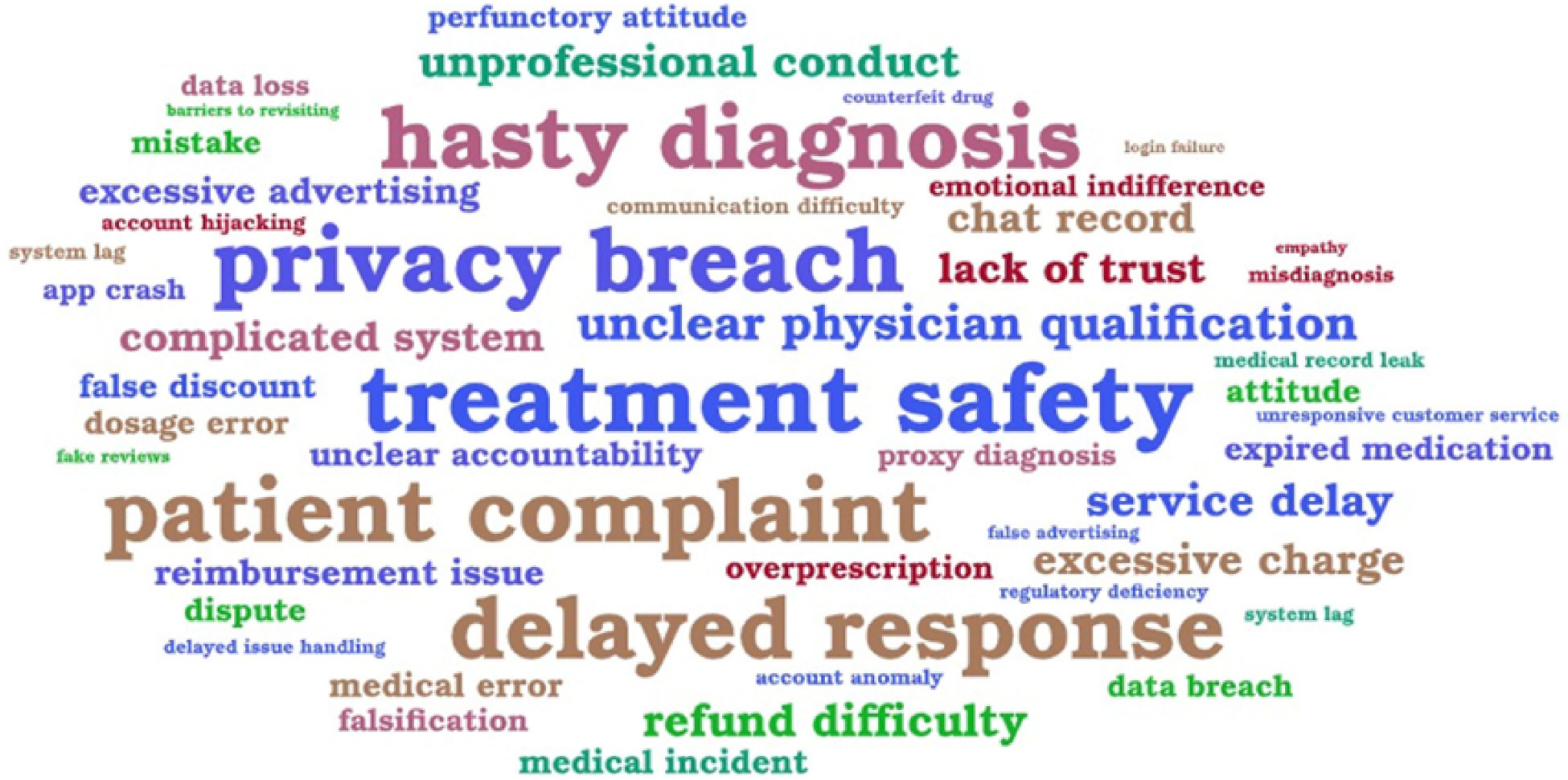
Word cloud of frequent risk-related terms mentioned by users.

As illustrated in Figure 3, the size and boldness of each word correspond to its frequency of occurrence, thereby revealing the most salient topics discussed by users. The most prominent terms, such as treatment safety, privacy breaches, patient complaints, delayed response, and hasty diagnosis, highlight recurring concerns about medical reliability, privacy protection, service timeliness, and communication quality. These lexical patterns represent preliminary discussion themes—such as privacy, diagnosis, cost, and communication—that later served as the empirical foundation for grounded theory coding and categorization.

The word cloud analysis served as a preliminary and exploratory tool rather than a formal analytical step. Identification of these high-frequency terms provided a data-driven starting point for theoretical abstraction. This guided researchers in reviewing the relevant literature and refining the conceptual understanding of patient-perceived risk, thereby bridging the transition from exploratory text mining to interpretive qualitative analysis. Analysis of the collected data was conducted following the three-step process of Strauss and Corbin's evolved grounded theory methodology (48), including open, axial, and selective coding, to construct a comprehensive framework of perceived risk in OHSs.

Open coding, the initial phase of coding, focused on identifying categories for each sentence to establish a conceptual meaning from the data (49). At this stage, all patient-generated textual data were systematically reviewed and analyzed. Expressions that reflected dissatisfaction, uncertainty, or perceived risk were extracted and labeled as initial concepts.

Through iterative comparisons and refinement, more than 2,000 raw statements were identified and subsequently condensed into a series of formal concepts, which were grouped into 22 preliminary categories based on their semantic similarities. This process transformed fragmented online comments into analytically meaningful categories and provided an empirical foundation for axial coding.

Table 2 presents three representative raw statements and their corresponding conceptual labels for each category.

**Table 2:** Examples of open coding: categories and Raw data excerpts.

Categories	Raw data excerpts
Diagnostic inaccuracy	The doctor misjudged my symptoms and gave the wrong advice./Without a physical exam, the diagnosis felt unreliable./I was told I had a minor issue, but later it turned serious.
Perfunctory consultation	The doctor only replied with a few words./It felt like the doctor didn't really read my messages carefully./The replies were very brief and lacked professional advice.
Delayed treatment	I waited for hours before the doctor replied./The delay in diagnosis made my condition worse./The follow-up messages came too late to be helpful.
Overprescription	The doctor prescribed too many unnecessary medications./I was given several drugs that didn't match my symptoms./They prescribed expensive medicines during the online session.
Medication safety	I'm not sure if the prescribed drug is suitable for my condition./The online doctor did not explain the side effects clearly./The medicine I received caused unexpected reactions.
Doctor qualification and authenticity	I was not sure whether the doctor was genuinely qualified or just using someone else's license./The platform displayed the doctor's profile, but there was no way to verify whether the information provided was accurate./ It felt like the doctor's identity was unclear.
Proxy consultation	The assistant answered my questions instead of the doctor./I realized the replies came from someone other than the listed physician./The doctor was too busy and delegated my case to another person.
Reimbursement limitation	Online prescriptions can't be reimbursed under my insurance plan./I had to pay all costs upfront and couldn't claim reimbursement./The doctor's receipt was not accepted by the insurance system.
Price opacity	The consultation price seemed to change frequently when I booked./There was no clear explanation for the extra service fees./The total charge was much higher than initially stated.
High medical expenses	The online consultation costs more than visiting the hospital in person./I spent nearly 300 RMB for just a few minutes of chatting./The platform added hidden fees I didn't notice at first.
Settlement difficulty	It was hard to complete the payment on the platform./Refunds and settlements took a long time to process./My refund request was ignored after the service ended.
Inaccessible complaint channels	I couldn't find where to file a complaint on the website./Customer service didn't respond to my complaint emails./The feedback system always showed “error” when I tried to report issues.
Ambiguous accountability	When something went wrong, neither the platform nor the doctor took responsibility./The doctor blamed the platform, and the platform blamed the doctor./There was no clear accountability for the treatment outcome.
Regulatory inadequacy	It feels like there's no oversight on these online doctors./I'm not sure whether these OHPs are legally monitored./There's no clear rule on how to handle patient disputes online.
System lag	The OHS app kept freezing during the session./I got disconnected multiple times while chatting with the doctor./The platform crashed when I tried to upload my report.
Cumbersome operation	It took me several steps just to upload one image./The interface was confusing and hard to use./I had to re-enter the same information multiple times.
Advertisement disturbance	Pop-up ads kept appearing during my consultation./Promotional messages covered the chat window./The page redirected me to unrelated product links.
Personal information disclosure	I received promotional calls after submitting my medical information online./My phone number was visible to others in the chat history./I'm worried that my consultation records can be accessed by strangers.
Excessive data collection	The platform asked for my full ID number just to book an appointment./Too many personal details were required before I could start the consultation./It felt invasive when the system asked for information unrelated to my illness.
Difficulty in information removal	I could not delete my past consultation records from the platform./Once uploaded, my medical reports stayed online with no option to remove them./I tried to hide my personal data, but there was no privacy control available.
Impersonal doctor–patient communication	The doctor's tone felt cold and indifferent throughout the chat./The replies were short and formulaic, like copy-pasted responses./It felt like I was talking to a system, not a real person.
Lack of emotional support	The doctor did not show any concern about my anxiety or worries./ I hoped for some comfort or understanding, but the response was emotionless./There was no encouragement or reassurance from the doctor at all.

Following the open coding phase, axial coding was conducted to identify relationships among the 22 preliminary categories and to organize them into broader conceptual clusters. Through iterative comparisons and thematic clustering, these categories were consolidated into eight higher-order dimensions. This process involved clustering categories with similar themes into broader subcategories based on their interconnections and logical order, and then consolidating them into main categories (50).

This analytical process revealed that the perceived risks in OHSs are multifaceted, encompassing the clinical, professional, financial, governance, technological, informational, and emotional domains. These dimensions include medical authenticity, prescription and medication safety, diagnostic quality, reimbursement limitations, platform accountability, technical performance, data privacy, and emotional support. Collectively, these parameters provide a comprehensive understanding of the complex perceived risk structure within an OHS. The relationships among the eight principal categories and their corresponding subcategories identified through axial coding are presented in Table 3.

**Table 3:** Main categories and corresponding subcategories identified through axial coding.

Main category	Subcategories	Conceptual description
Medical authenticity and professionalism	Doctor qualification and authenticity; Proxy consultation	Reflects concerns about the authenticity of doctors and whether consultations are conducted by qualified professionals.
Prescription and medication management	Overprescription; Medication safety	Involves issues of excessive prescriptions, inappropriate medication, and potential drug safety problems.
Diagnostic quality and efficiency	Diagnostic inaccuracy; Perfunctory consultation; Delayed treatment	Refers to diagnostic errors, perfunctory responses, and untimely medical advice.
Financial and reimbursement barriers	Reimbursement limitation; Price opacity; High medical expense; Settlement difficulty	Captures patients’ difficulties in understanding costs, reimbursement restrictions, and payment procedures.
Platform and accountability	Inaccessible complaint channels; Ambiguous accountability; Regulatory inadequacy	Highlights the lack of clear responsibility, oversight, and accessible complaint systems.
Technical performance and interaction	System lag; Cumbersome operation; Advertisement disturbance	Describes technological instability, system lag, and inefficient data communication.
Data privacy and information security	Personal information disclosure; Excessive data collection; Difficulty in information removal	Encompasses concerns about data exposure, unauthorized collection, and limited control over personal information.
Emotional support and communication quality	Impersonal doctor–patient communication; Lack of emotional support	Focuses on the emotional distance and lack of empathy or psychological comfort in online consultations.

In the selective coding final step, we refined and verified the relationships between the main categories and subcategories derived from the axial coding phase. This process involved identifying a core category that conceptually integrated all other dimensions and provided a theoretical explanation for their relationships. The purpose of this process is to explore the relationships between the core and main categories in depth and explain all phenomena or events with reference to a coherent storyline (45). Selective coding represents the culmination of a grounded theory analysis, in which fragmented categories are unified into an overarching analytical narrative. It not only consolidates the results of open and axial coding but also transforms descriptive findings into a theoretical model that captures the underlying logic of the observed phenomena.

Through iterative comparison and theoretical abstraction, the core category of this study was identified as the “perceived safety–trust.” This concept captures how patients continuously balance perceptions of safety (“Is this service reliable and risk-free?”) and trust (“Is this service credible and worth relying on?”) within OHSs. Each of the eight major categories perturbs this equilibrium through distinct mechanisms, collectively influencing patient confidence in OHPs and their willingness to continue using them.

**Figure 4:**
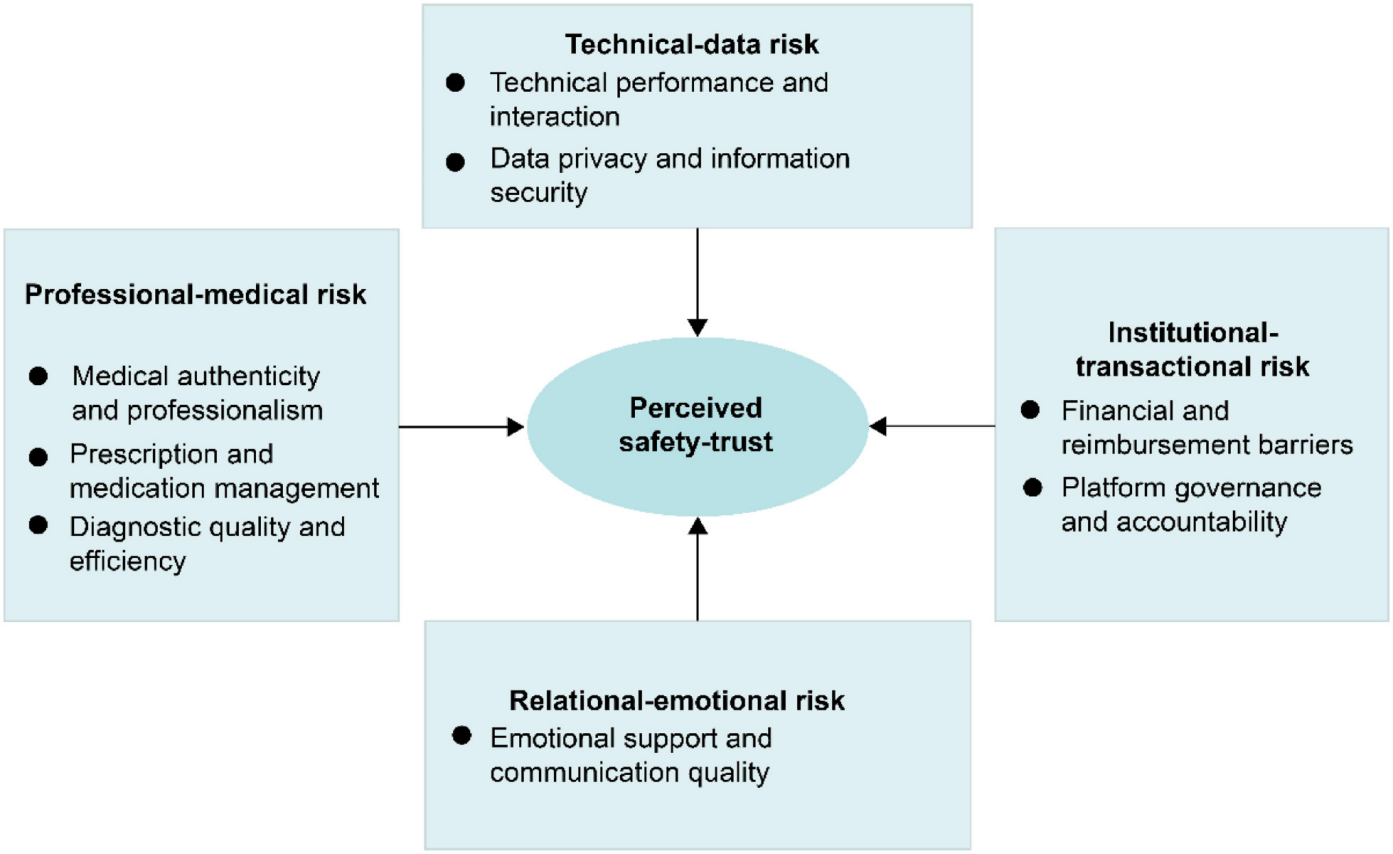
The perceived safety–trust model in OHSs.

To conceptualize this dynamic process, the eight categories were further synthesized into four interrelated layers that jointly shaped patients’ perceptions of safety and trust (Figure 4). The professional medical layer ensures clinical competence and diagnostic reliability, thereby forming the cognitive foundation of professional trust. The institutional-transactional layer provides procedural fairness and accountability, reinforcing confidence in OHSs’ regulatory and financial environments. The technical data layer safeguards digital stability and information control, underpinning patient confidence in the technological infrastructure for care delivery. Finally, the relational-emotional layer conveys empathy and interpersonal reassurance and transforms cognitive trust into affective trust through emotional resonance.

These four layers are not independent but are dynamically interwoven. Professional competence and institutional assurance form the structural foundation of perceived safety, while technological reliability and emotional connections act as catalysts that sustain and amplify trust. When these elements operate synergistically, patients perceive OHSs as reliable and humane; however, disruptions in any dimension can cascade across others, amplifying perceived risks and undermining the overall safety trust model.

### 3.3 Changes in perceived risk over time

**Figure 5:**
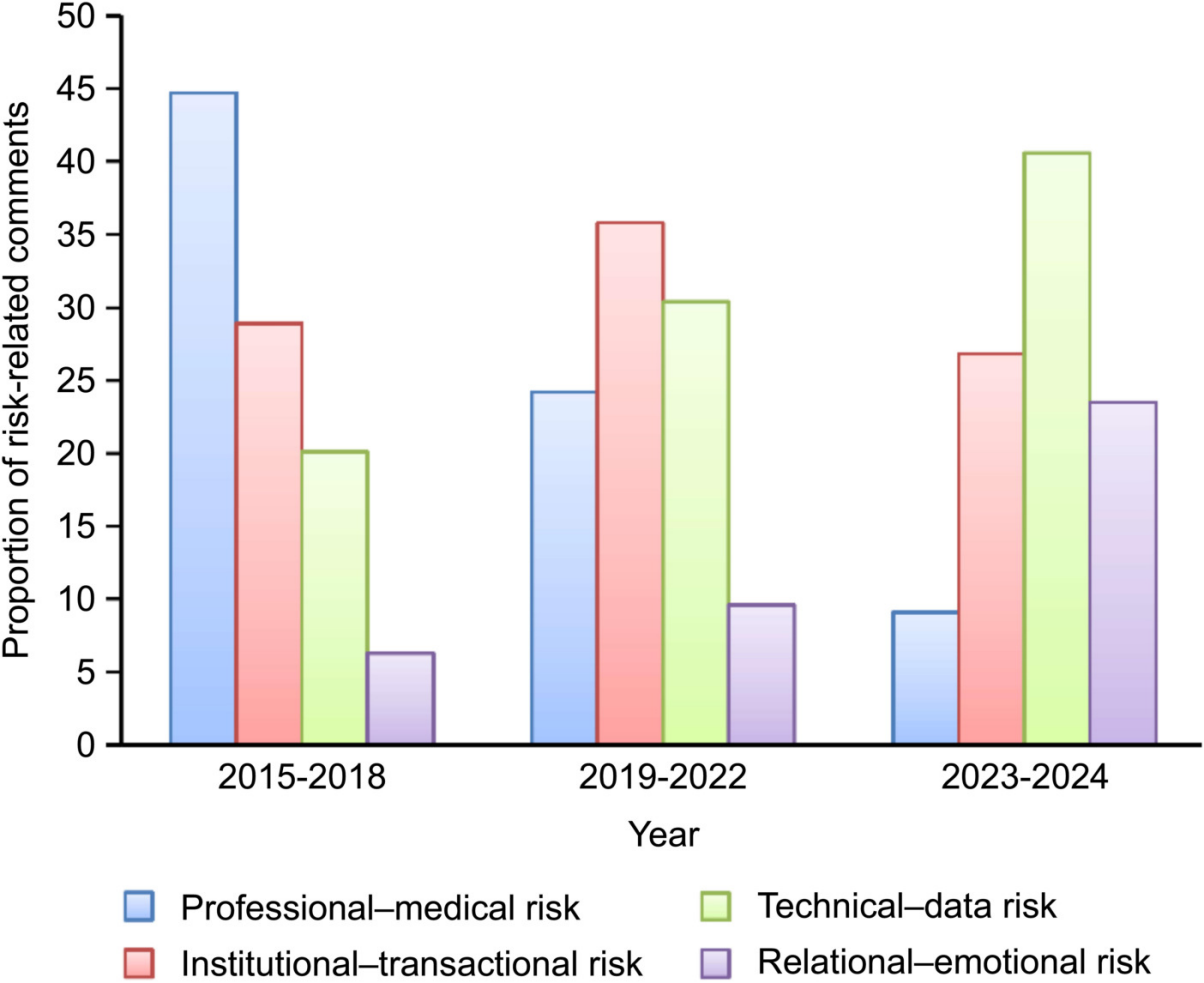
Temporal evolution of perceived risk in OHSs from 2015 to 2024.

Figure 5 illustrates the temporal shifts in the patient-perceived risk dimensions across the three stages of online healthcare development. During the early stage (2015–2018), perceived risk was dominated by professional medical risks, such as physician authenticity, diagnostic accuracy, and prescription reliability. At this stage, patients primarily evaluated whether OHSs could provide trustworthy medical care. Institutional-transactional risk ranked second, reflecting concerns about unclear pricing and limited reimbursement mechanisms, while technical–data and relational–emotional risks were less prominent.

During the middle stage (2019–2022), the perceived risk landscape changed significantly. Institutional-transactional risk became the most salient, as the large-scale adoption of online consultations exposed governance and accountability gaps in platform regulation and financial settlement. The importance of technical data risk has increased because of massive digital traffic, platform congestion, and privacy protection challenges. Meanwhile, professional-medical risk declined moderately as online healthcare gained legitimacy, while relational-emotional risk rose slightly, reflecting emerging expectations for empathy and communication quality.

By the later stage (2023–2024), patient concerns further evolved toward technical data risk, which is now the most critical risk domain. Data security, system stability, and digital transparency are the decisive factors shaping perceived safety. Simultaneously, relational-emotional risk rose to second place, underscoring the growing importance of emotional reassurance and human connections in digital health interactions. Conversely, institutional-transactional and professional-medical risks declined, reflecting the institutionalization and standardization of OHSs.

This temporal trajectory illustrates a gradual shift in patients’ perceived risks—from medical professionalism–centered concerns during the early stage to technology- and relationship-centered concerns in the post-pandemic stage. Over time, patient trust in OHSs has become increasingly shaped by digital reliability and relational warmth rather than solely by traditional clinical credibility. This transformation indicates that the foundations of trust have evolved from professional competence to technological assurance and emotional connections.

At the same time, it is important to reflect on the potential influence of changes in comment composition on the observed temporal patterns. Although Figure 5 presents the relative distribution of perceived risk dimensions rather than absolute comment counts, these proportional changes may still be affected by shifts in how user comments are distributed across risk categories over time. Such structural variation is partly related to platform expansion, diversification of user participation, and broader behavioral transformations in online health engagement. In this context, some observed changes—particularly the declining proportion of professional-medical risk—may be associated with a reduced relative emphasis on early-stage credibility concerns, as user attention gradually shifted toward more complex technological and relational issues.

Importantly, changes in comment composition cannot be assumed to be evenly distributed across all risk types. As baseline trust in OHSs improved, concerns related to professional-medical risk may have occupied a smaller share of user discussions. In contrast, technical–data and relational–emotional risks likely accounted for a growing proportion of comments due to increased awareness of data security, system reliability, and service experience.

Taken together, the observed shifts in relative risk salience can be meaningfully interpreted in relation to broader institutional, technological, and behavioral transformations shaping the development of OHSs, including regulatory evolution, digital infrastructure expansion, and changing patient expectations.

## 4 Discussion

### 4.1 Principal findings

The findings of this study address the three research objectives outlined in the Introduction and offer a systematic account of perceived risks in OHSs. More importantly, the results provide insight into why these perceptions are structured and evolve in the ways observed.

First, we identified 22 initial categories of perceived risk that were consolidated into eight main categories. These categories were further integrated into four interrelated risk domains—professional-medical risk, institutional-transactional risk, technical-data risk, and relational-emotional risk—which together constitute a perceived safety–trust model.

Second, the four domains capture distinct experiential patterns: professional-medical risk arises from inadequate credibility indicators; institutional-transactional risk from unclear responsibilities and opaque procedures; technical-data risk from system instability and privacy intrusions; and relational-emotional risk from impersonal or unresponsive communication. These domains are systemically interconnected—deficiencies in one can amplify risks in others, while improvements can buffer risks elsewhere.

Third, the configuration of perceived risk exhibited a distinct temporal evolution. During the early stage of OHS adoption, concerns centered on professional-medical risk (e.g., diagnostic accuracy, physician authenticity, and prescription reliability) reflected uncertainty about whether online consultations could provide trustworthy clinical care. As platforms expanded and became institutionalized, attention shifted to institutional-transactional and technical-data risks, including price opacity, ambiguous accountability, and privacy and data protection vulnerabilities. Recently, patient attention has been increasingly focused on technological reliability and relational assurance, suggesting that the foundation of trust has broadened beyond clinical competence to include digital assurance and emotional connectedness. This temporal shift underscores the evolution of patient perceptions in parallel with the technological maturity and normalization of OHSs.

Finally, the present findings primarily reflect OHS user perceptions. Non-users may hold higher baseline risk perceptions regarding data security, professional authenticity, or technological dependence, which may discourage initial adoption. This suggests that observed patterns are shaped not only by service experience but also by selection processes, whereby individuals with higher trust thresholds or greater digital familiarity are more likely to engage with OHSs.

### 4.2 Comparison with prior literature

It is worthwhile to compare the four risk domains identified in this study with the existing literature on OHSs.

Regarding professional-medical risk, our finding that concerns about physician identity and diagnostic quality dominate early-stage perceptions is consistent with prior evidence (33, 51). A study conducted in Germany found that the highest perceived risks of telemedicine were related to potential misdiagnosis and concerns about the inability to establish the same patient-physician connection as in face-to-face care (52). Similarly, Liu et al. confirmed that doctors’ credibility exerts the strongest positive effect on online patient trust (53). Our findings further extend this literature by demonstrating that professional-medical risk is not a static barrier but a stage-specific concern that diminishes in salience as users accumulate experiential familiarity with digital consultation formats.

With respect to institutional-transactional risk, trust in healthcare organizations and institutional mechanisms positively influences telemedicine acceptance (54, 55), while institutional guarantees further enhance online patient trust (53). Within this domain, financial risk constitutes a key dimension of institutional-transactional risk and has been identified as a significant adoption barrier, particularly among lower-income groups in Taiwan (56). Taken together, these studies and our findings suggest that perceived institutional-transactional risk reflects not merely economic concerns but deeper anxieties about governance transparency and procedural justice in platform-mediated healthcare environments.

The technical-data risk domain is particularly relevant given escalating global concerns about digital health privacy. Technical performance and privacy control have been established as critical antecedents of perceived risk in OHSs (57, 58). Kuen et al. demonstrated that privacy risk reduces telemedicine use intention, whereas trust in technology indirectly mitigates perceived risk via trust transfer to treatment (34). Our analysis further indicates that technical-data risk has become increasingly salient over time and now constitutes one of the most prominent dimensions of perceived risk in OHSs. This temporal transition suggests that as baseline professional trust stabilizes, users recalibrate their risk assessments toward infrastructural and data-related vulnerabilities embedded in platform-based healthcare systems.

The relational-emotional risk domain captures an experiential pattern that has received limited theoretical attention. Digital trust research has underscored the role of emotional resonance in patient-provider communication (59, 60). While recent studies have documented that physicians’ emotional support positively influences patient adoption of online consultations and that psychological discomfort during telemedicine encounters reduces usage intention, these observations have typically been subsumed under broader constructs such as “trust risk” or “psychological risk” (55, 56). By contrast, our study identifies relational-emotional risk as a distinct experiential domain that interacts dynamically with clinical, institutional, and technical dimensions.

### 4.3 Strengths and limitations

#### 4.3.1 Theoretical implications

This study makes two theoretical contributions. First, by integrating web crawling technology with grounded theory methodology, this study collected and analyzed large-scale naturalistic user-generated data from OHP, and inductively identified a four-domain perceived risk structure—professional-medical, institutional-transactional, technical-data, and relational-emotional. Unlike prior studies that predominantly relied on deductive, survey-based approaches, the data-driven strategy adopted here allowed risk categories to emerge directly from authentic user experiences.

Second, this study moves beyond a static description of risk categories to map the temporal evolution of perceived risk across different stages of OHSs development. The findings reveal that risk perceptions shift systematically. This dynamic trajectory challenges the prevailing treatment of perceived risk as a fixed pre-adoption barrier and demonstrates that risk appraisal co-evolves with the maturation of digital health ecosystems.

#### 4.3.2 Practical implications

The findings offer actionable implications for practitioners and policymakers. For OHP operators and healthcare providers, the four-domain risk structure highlights the necessity of adopting a holistic approach to risk management. OHP should implement integrated quality assurance systems that simultaneously cover physician credentialing, transparent fee structures, stable technical infrastructure, and humanized communication protocols. The systemic interconnections among the four domains suggest that investing in technical stability and governance transparency can buffer occasional clinical shortcomings, while empathetic communication can mitigate user frustration arising from technical disruptions.

For policymakers, this study underscores the need for comprehensive regulatory frameworks that extend beyond clinical standards and data privacy to encompass platform governance, including fee transparency, liability allocation, and accessible dispute resolution. The dynamic evolution of perceived risk revealed in this study indicates that regulatory frameworks should be periodically updated as digital health ecosystems evolve.

#### 4.3.3 Limitations

There are limitations to this study that need to be acknowledged. First, web crawling is based on data sourced from four major Chinese online forums, which, while large and diverse, may not fully represent all OHSs users or other cultural contexts. Future studies could examine the generalizability of the perceived safety–trust model across different countries and healthcare systems. Second, although the grounded theory provided deep conceptual insights, the analysis relied solely on qualitative interpretation, without quantitative validation. Future work may adopt mixed-method designs or structural equation modeling to test the relationships among the identified domains and to assess their predictive validity. Third, as noted above, the findings primarily reflect the perceptions of OHSs users; non-users who may hold higher baseline risk perceptions remain unrepresented. Future research should integrate multisource data, such as surveys combining both users and non-users, to examine how perceived risks vary across user subgroups and to understand the anticipatory risk perceptions that may discourage initial adoption.

## 5 Conclusions

This study progressively identified and refined the dimensions of perceived risk in OHSs, providing an integrated explanation of how different risk dimensions function and interact in digital healthcare settings. We report that through four interrelated domains—clinical-professional, institutional-transactional, technical-data, and relational-affective— perceived risks arise from the dynamic interplay between professional credibility, institutional assurance, technological reliability, and emotional responsiveness. The perceived safety–trust model developed in this study serves as a practical framework for improving OHP governance. It emphasizes that fostering user trust requires not only professional and technical competence, but also transparent institutional mechanisms and empathetic communication. As digital healthcare continues to expand and integrate emerging technologies, maintaining system reliability while preserving the warmth of human communication is crucial to sustaining patient confidence and building equitable, trustworthy, and patient-centered online healthcare environments.

## Data Availability

The original contributions presented in the study are included in the article/Supplementary Material, further inquiries can be directed to the corresponding author.

## References

[1] Aceto G Persico V Pescapé A. Industry 4.0 and health: internet of things, big data, and cloud computing for healthcare 4.0. J Ind Inf Integr. (2020) 18:100129. 10.1016/j.jii.2020.100129

[2] Mizrachi Y Shahrabani S Nachmani M Hornik A. Obstacles to using online health services among adults age 50 and up and the role of family support in overcoming them. Isr J Health Policy Res. (2020) 9:42. 10.1186/s13584-020-00398-x 32825840 PMC7441221

[3] Jiang X Xie H Tang R Du Y Li T Gao J Characteristics of online health care services from China’s largest online medical platform: cross-sectional survey study. J Med Internet Res. (2021) 23:e25817. 10.2196/25817 33729985 PMC8051434

[4] Chauhan A Jakhar SK Jabbour CJC. Implications for sustainable healthcare operations in embracing telemedicine services during a pandemic. Technol Forecast Soc Change. (2022) 176:121462. 10.1016/j.techfore.2021.121462 35034990 PMC8743184

[5] The 55th Statistical Report on Internet Development in China. China Internet Network Information Center. (2025). Available online at: https://www.cnnic.net.cn/n4/2025/0117/c88-11229.html (Accessed March 25, 2025).

[6] White Paper on Online Healthcare Service Consumption in China (Brief Edition). iResearch Consulting Group; JD Health (2022). Available online at: https://www.idigital.com.cn/report/detail?id=4057 (Accessed October 10, 2024).

[7] Lee SM Lee DH. Opportunities and challenges for contactless healthcare services in the post-COVID-19 era. Technol Forecast Soc Change. (2021) 167:120712. 10.1016/j.techfore.2021.120712 33654330 PMC7908833

[8] Zhuo X Wang WT. Why are physicians willing to contribute knowledge? Evidence from online health communities. Comput Hum Behav. (2024) 152:108095. 10.1016/j.chb.2023.108095

[9] Tu J Wang C Wu S. The internet hospital: an emerging innovation in China. Lancet Glob Health. (2015) 3:e445–6. 10.1016/s2214-109x(15)00042-x 26187488 PMC7129805

[10] Johnston AC Worrell JL Di Gangi PM Wasko M. Online health communities: an assessment of the influence of participation on patient empowerment outcomes. Inf Technol People. (2013) 26:213–35. 10.1108/ITP-02-2013-0040

[11] Ding L She Q Chen F Chen Z Jiang M Huang H The internet hospital plus drug delivery platform for health management during the COVID-19 pandemic: observational study. J Med Internet Res. (2020) 22:e19678. 10.2196/19678 32716892 PMC7419153

[12] Senek M Drummond D Pinnock H Hansen K Ankolekar A O’Connor Ú Impact of digital health on patient-provider relationships in respiratory secondary care based on qualitative and quantitative evidence: systematic review. J Med Internet Res. (2025) 27:e70970. 10.2196/70970 40446293 PMC12166327

[13] Yang Y Zhang X Lee PKC. Improving the effectiveness of online healthcare platforms: an empirical study with multi-period patient–doctor consultation data. Int J Prod Econ. (2019) 207:70–80. 10.1016/j.ijpe.2018.11.009

[14] Lattie EG Adkins EC Winquist N Stiles-Shields C Wafford QE Graham AK. Digital mental health interventions for depression, anxiety, and enhancement of psychological well-being among college students: systematic review. J Med Internet Res. (2019) 21:e12869. 10.2196/12869 31333198 PMC6681642

[15] Livieri G Mangina E Protopapadakis ED Panayiotou AG. The gaps and challenges in digital health technology use as perceived by patients: a scoping review and narrative meta-synthesis. Front Digit Health. (2025) 7:1474956. 10.3389/fdgth.2025.1474956 40212901 PMC11983460

[16] Anderson J Walsh J Anderson M Burnley R. Patient satisfaction with remote consultations in a primary care setting. Cureus. (2021) 13:e17814. 10.7759/cureus.17814 34660024 PMC8498974

[17] Schutz S Walthall H Snowball J Vagner R Fernandez N Bartram E Patient and clinician experiences of remote consultation during the SARS-CoV-2 pandemic: a service evaluation. Digit Health. (2022) 8:20552076221115022. 10.1177/20552076221115022 35959197 PMC9358347

[18] Ewoh P Vartiainen T. Vulnerability to cyberattacks and sociotechnical solutions for health care systems: systematic review. J Med Internet Res. (2024) 26:e46904. 10.2196/46904 38820579 PMC11179043

[19] Belfrage S Helgesson G Lynøe N. Trust and digital privacy in healthcare: a cross-sectional descriptive study of trust and attitudes towards uses of electronic health data among the general public in Sweden. BMC Med Ethics. (2022) 23:19. 10.1186/s12910-022-00758-z 35246118 PMC8896318

[20] Hong Z Deng Z Zhang W. Examining factors affecting patients trust in online healthcare services in China: the moderating role of the purpose of use. Health Informatics J. (2019) 25:1647–60. 10.1177/1460458218796660 30192694

[21] Mitchell VW. Understanding consumers’ behaviour: can perceived risk theory help? Manag Decis. (1992) 30. 10.1108/00251749210013050

[22] Bauer RA. Consumer behavior as risk raking. In: Hancock R, editor. Dynamic Marketing for a Changing World. Chicago: American Marketing Association (1960). p. 389–98.

[23] Dowling GR Staelin R. A model of perceived risk and intended risk-handling activity. J Consum Res. (1994) 21:119–34. 10.1086/209386

[24] Jacoby J Kaplan LB. The components of perceived risk. In: Venkatesan M, editor. Advances in Consumer Research. Chicago, Ill: Association for Consumer Research (1972). p. 382–93.

[25] Peter JP Tarpey LX. A comparative analysis of three consumer decision strategies. J Consum Res. (1975) 2:29–37. 10.1086/208613

[26] Featherman MS Pavlou PA. Predicting e-services adoption: a perceived risk facets perspective. Int J Hum-Comput Stud. (2003) 59:451–74. 10.1016/S1071-5819(03)00111-3

[27] Deng Z Hong Z Ren C Zhang W Xiang F. What predicts patients’ adoption intention toward mHealth services in China: empirical study. JMIR mHealth UHealth. (2018) 6:e172. 10.2196/mhealth.9316 30158101 PMC6135967

[28] Fischer SH David D Crotty BH Dierks M Safran C. Acceptance and use of health information technology by community-dwelling elders. Int J Med Inform. (2014) 83:624–35. 10.1016/j.ijmedinf.2014.06.005 24996581 PMC4144164

[29] Rasche P Wille M Bröhl C Theis S Schäfer K Knobe M Prevalence of health app use among older adults in Germany: national survey. JMIR mHealth UHealth. (2018) 6:e26. 10.2196/mhealth.8619 29362211 PMC5801520

[30] Parker SJ Jessel S Richardson JE Reid MC. Older adults are mobile too! identifying the barriers and facilitators to older adults’ use of mHealth for pain management. BMC Geriatr. (2013) 13:43. 10.1186/1471-2318-13-43 23647949 PMC3673892

[31] Campbell K Greenfield G Li E O’Brien N Hayhoe B Beaney T The impact of virtual consultations on the quality of primary care: systematic review. J Med Internet Res. (2023) 25:e48920. 10.2196/48920 37647117 PMC10500356

[32] O’Malley G Shaikh U Marcin JP. Telehealth and Patient Safety. PSNet. Rockville, Md: Agency for Healthcare Research and Quality, United States Department of Health and Human Services (2022). Available online at: https://psnet.ahrq.gov/primer/telehealth-and-patient-safety?utm_source=chatgpt.com (Accessed February 25, 2025).

[33] Klaver NS Van de Klundert J van den Broek RJGM Askari M. Relationship between perceived risks of using mHealth applications and the intention to use them among older adults in The Netherlands: cross-sectional study. JMIR mHealth UHealth. (2021) 9:e26845. 10.2196/26845 34459745 PMC8438611

[34] Kuen L Schürmann F Westmattelmann D Hartwig S Tzafrir S Schewe G. Trust transfer effects and associated risks in telemedicine adoption. Electron Markets. (2023) 33:35. 10.1007/s12525-023-00657-0

[35] Guo X Zhang X Sun Y. The privacy–personalization paradox in mHealth services acceptance of different age groups. Electron Commer Res Appl. (2016) 16:55–65. 10.1016/j.elerap.2015.11.001

[36] Zhao Y Ni Q Zhou R. What factors influence the mobile health service adoption? A meta-analysis and the moderating role of age. Int J Inf Manage. (2018) 43:342–50. 10.1016/j.ijinfomgt.2017.08.006

[37] Wang Z Chen H Luo J Wang C Xu X Zhou Y. Sharing service in healthcare systems: a recent survey. Omega (Westport). (2024b) 129:103158. 10.1016/j.omega.2024.103158

[38] Wang L Liang D HuangFu H Ke C Wu S Lai Y. Enterprise-led internet healthcare provision in China: insights from a leading platform. Front Digit Health. (2025) 7:1491183. 10.3389/fdgth.2025.1491183 40099033 PMC11911379

[39] Han T Wei Q Wang R Cai Y Zhu H Chen J Service quality and patient satisfaction of internet hospitals in China: cross-sectional evaluation with the service quality questionnaire. J Med Internet Res. (2024) 26:e55140. 10.2196/55140 39514849 PMC11584541

[40] Cheng X Zhang S Fan Q Sun X Zhang X. How to guarantee online service quality of physicians on the health platforms? Ind Manag Data Syst. (2025) 125(11): 2978–2998. 10.1108/IMDS-10-2024-1010

[41] Wissawaswaengsuk P Kumar P Frank B Badir YF. The role of trust as the facilitator and contingency factor in the adoption of digital healthcare services: a telemedicine context. Comput Hum Behav. (2025) 172:108722. 10.1016/j.chb.2025.108722

[42] Li J Chu C Zhang J Sun J. Bilateral pricing for internet healthcare platform: separating or pooling doctors? Expert Syst Appl. (2025) 294:128717. 10.1016/j.eswa.2025.128717

[43] Mohajan D Mohajan HK. Straussian grounded theory: an evolved variant in qualitative research. SSSH. (2023) 2:33–40. 10.56397/SSSH.2023.02.06

[44] Corbin JM Strauss A. Grounded theory research: procedures, canons, and evaluative criteria. Qual Sociol. (1990) 13:3–21. 10.1007/BF00988593

[45] de la Espriella R Gómez Restrepo C. Grounded theory. Rev Colomb Psiquiatr (Engl Ed). (2020) 49:127–33. 10.1016/j.rcp.2018.08.002 32446420

[46] Strauss AL Corbin JM. Basics of Qualitative Research: Techniques and Procedures for Developing Grounded Theory. 2nd ed Thousand Oaks, Calif: Sage Publications (1998).

[47] Fassinger RE. Paradigms, praxis, problems, and promise: grounded theory in counseling psychology research. J Couns Psychol. (2005) 52:156–66. 10.1037/0022-0167.52.2.156

[48] Chun Tie Y Birks M Francis K. Grounded theory research: a design framework for novice researchers. Sage Open Med. (2019) 7:2050312118822927. 10.1177/2050312118822927 30637106 PMC6318722

[49] Strauss A Corbon JJ. Basics of Qualitative Research: Grounded Theory Procedures and Techniques. Newbury Park: Sage Publications (1990). p. 53–5.

[50] Zhang JX Cheng JW Philbin SP Ballesteros-Perez P Skitmore M Wang G. Influencing factors of urban innovation and development: a grounded theory analysis. Environ Dev Sustain. (2023) 25:2079–104. 10.1007/s10668-022-02151-7 35125938 PMC8809240

[51] Wu TC Ho CT. Reconstructing risk dimensions in telemedicine: investigating technology adoption and barriers during the COVID-19 pandemic in Taiwan. J Med Internet Res. (2025) 27:e53306. 10.2196/53306 39899842 PMC11833260

[52] Weißenfeld MM Goetz K Steinhäuser J. Facilitators and barriers for the implementation of telemedicine from a local government point of view: a cross-sectional survey in Germany. BMC Health Serv Res. (2021) 21:919. 10.1186/s12913-021-06929-9 34488753 PMC8419374

[53] Liu C Wang J Chen R Zhou W. Exploring the influence of Chinese online patient trust on telemedicine behavior: insights into perceived risk and behavior intention. Front Public Health. (2024) 12:1415889. 10.3389/fpubh.2024.1415889 39247232 PMC11377225

[54] Catapan SC Sazon H Zheng S Gallegos-Rejas V Mendis R Santiago PHR A systematic review of consumers’ and healthcare professionals’ trust in digital healthcare. NPJ Digit Med. (2025) 8:115. 10.1038/s41746-025-01510-8 39984678 PMC11845731

[55] Bahari G Mutambik I Almuqrin A Alharbi ZH. Trust: how it affects the use of telemedicine in improving access to assistive technology to enhance healthcare services. Risk Manag Healthc Policy. (2024) 17:1859–73. 10.2147/rmhp.S469324 39072188 PMC11283829

[56] Wu TC Ho CB. Barriers to telemedicine adoption during the COVID-19 pandemic in Taiwan: comparison of perceived risks by socioeconomic status correlates. Int J Environ Res Public Health. (2023) 20:3504. 10.3390/ijerph20043504 36834205 PMC9966241

[57] Kalckreuth V Feufel N A M. Extending the privacy calculus to the mHealth domain: survey study on the intention to use mHealth apps in Germany. JMIR Hum Factors. (2023) 10:e45503. 10.2196/45503 37585259 PMC10468710

[58] Atalay HN Yücel Ş. Decoding privacy concerns: the role of perceived risk and benefits in personal health data disclosure. Arch Public Health. (2024) 82:180. 10.1186/s13690-024-01416-z 39394170 PMC11468474

[59] Rui JR Guo J Yang K. How do provider communication strategies predict online patient satisfaction? A content analysis of online patient-provider communication transcripts. Digit Health. (2024) 10:20552076241255617. 10.1177/20552076241255617 38778866 PMC11110499

[60] Abou Hashish EA. Compassion through technology: digital empathy concept analysis and implications in nursing. Digit Health. (2025) 11:20552076251326221. 10.1177/20552076251326221 40093701 PMC11907611

